# Supplementary material for: let-7 coordinates the transition to adulthood through a single primary and four secondary targets
Source: Life Sci Alliance. 2019 Mar 25;2(2):e201900335. doi: 10.26508/lsa.201900335 (PMC6435043; doi:10.26508/lsa.201900335)
Supplement: Supplementary file 5 [file LSA-2019-00335_TableS5.doc]

| Identifier / Strain number | Genotype | Source |
| --- | --- | --- |
| N2 | Wild-type | CGC |
| [MT7626](https://cgc.umn.edu/strain/MT7626) | *let-7(n2853)* X | CGC, (Reinhart et al., 2000) |
| HW1870 | *lin-41(xe8) / lin-41(bch28[Peft-3::gfp::h2b::tbb-2 3'UTR] xe70)* I | This study |
| HW1330 | *lin-41(xe11)* I; *let-7(n2853)* X | (Ecsedi et al., 2015) |
| HW1329 | *lin-41(xe11)* I | (Ecsedi et al., 2015) |
| HW1692 | *lin-29(xe37)* II | This study |
| HW1695 | *lin-29a(xe40)* II | This study |
| HW1698 | *mab-10(xe44)* II | This study |
| HW2384 | *mab-3(xe49)* II; *dmd-3(ok1327)* V | This study |
| HW1790 | *mab-10(xe44) lin-29(xe37) /* mnC1 II | This study |
| HW1758 | *mab-10(xe44) lin-29(xe40) /* mnC1 II | This study |
| HW2483 | *mab-3(xe49) mab-10(xe44) lin-29(xe40)* / mnC1 II; *dmd-3(ok1327)* V | This study |
| HW2382 | *lin-29a(xe40)* II; *let-7(n2853)* X | This study |
| HW2479 | *mab-10(xe44)* II; *let-7(n2853)* X | This study |
| HW2478 | *mab-10(xe44) lin-29a(xe40)* II; *let-7(n2853)* X | This study |
| HW2380 | *lin-29(xe37)* II; *let-7(n2853)* X | This study |
| SX346 | *unc119(e2598) III; wIs54[scm::gfp] V; lin-15(n765) X; mjIs15[ajm-1::mCherry]* | (Lehrbach et al., 2009) |
| HW1387 | *wIs54[scm::gfp]* V; *mjIs15[ajm-1::mCherry]*; *let-7(n2853)* X | This study |
| HW1865 | *lin-41(xe8) / lin-41(bch28 xe70)* I; *wIs54[scm::gfp]* V; *mjIs15[ajm-1::mCherry]* | This study |
| HW1922 | *lin-41(xe11)* I; *wIs54[scm::gfp]* V; *let-7(n2853)* X; *mjIs15[ajm-1::mCherry]* | This study |
| HW1923 | *lin-41(xe11)* I; *wIs54[scm::gfp]* V; *mjIs15[ajm-1::mCherry]* | This study |
| HW1861 | *lin-29a(xe40)* II; *wIs54[scm::gfp]* V; *mjIs15[ajm-1::mCherry]* | This study |
| HW1862 | *mab-10(xe44)* II; *wIs54[scm::gfp]* V; *mjIs15[ajm-1::mCherry]* | This study |
| HW1864 | *mab-10(xe44)* *lin-29a(xe40)* / mnC1 II; *wIs54[scm::gfp]* V; *mjIs15[ajm-1::mCherry]* | This study |
| HW2460 | *mab-3(xe49)* II; *dmd-3(ok1327)* *wIs54[scm::gfp]* V; *mjIs15[ajm-1::mCherry]* | This study |
| HW1508 | *him-5(e1490)* V | (Hodgkin et al., 1979) |
| HW1618 | *him-5(e1490)* V; *let-7(n2853)* X | This study |
| HW2572 | *lin-41(xe8) / lin-41(bch28 xe70)* I; *him-5(e1490)* V | This study |
| HW1616 | *lin-41(xe11)* I; *him-5(e1490)* V | This study |
| HW1617 | *lin-41(xe11)* I; *him-5(e1490)* V; *let-7(n2853)* X | This study |
| EM101 | *lin-41(bx37)* I; *him-5(e1490)* V | CGC, (Del Rio-Albrechtsen et al., 2006) |
| EM106 | *lin-41(bx42)* I; *him-5(e1490)* V | CGC, (Del Rio-Albrechtsen et al., 2006) |
| HW1829 | *lin-41(ma104)* I; *him-5(e1490)* V | This study |
| HW2385 | *mab-3(xe49)* II; *him-5(e1490) dmd-3(ok1327)* V | This study |
| HW1961 | *mab-10(xe44) lin-29a(xe40) /* mnC1 II; *him-5(e1490)* V | This study |
| HW1803 | *xeSi172 [Pmab-3::GFP(PEST)-H2B::mab-3 3'UTR]* II; *him-5(e1490)* V | This study |
| HW1798 | *xeSi181 [Pmab-3::GFP(PEST)-H2B::unc-54 3'UTR]* II; *him-5(e1490)* V | This study |
| HW1827 | *xeSi257 [Pdmd-3::GFP(PEST)-H2B::dmd-3 3'UTR]* II; *him-5(e1490)* V | This study |
| HW1828 | *xeSi255 [Pdmd-3::GFP(PEST)-H2B::unc-54 3'UTR]* II; *him-5(e1490)* V | This study |
| HW1801 | *lin-41(n2914)* I; *xeSi197[Plin-41::flag::gfp::lin-41::lin-41 3'UTR]* II, *him-5(e1490)* V | This study |
| HW1799 | *xeSi55[Pdpy-30::sart-3::gfp::his::flag::xrn-2 3'UTR]* I; *him-5(e1490)* V | This study |
| HW1826 | *lin-29(xe63[gfp::3xflag::lin-29a])* II | This study |
| HW1882 | *lin-29(xe63[gfp::3xflag::lin-29a])* II; *let-7(n2853)* X | This study |
| HW2295 | *xeSi417 [delta pes-10 minimal promoter and enhancer ACEL (lin-3)::FLAG-HA-degron::LIN-29A::lin-29 3'UTR::operon linker (SL2) with GFP-H2B::tbb-2 3'UTR]* II | This study |
| HW2321 | *xeSi417 [delta pes-10 minimal promoter and enhancer ACEL (lin-3)::FLAG-HA-degron::LIN-29A::lin-29 3'UTR::operon linker (SL2) with GFP-H2B::tbb-2 3'UTR]* *mab-10(xe44) lin-29a(xe40)* II / mnC1 | This study |
| HW2323 | *xeSi417 [delta pes-10 minimal promoter and enhancer ACEL (lin-3)::FLAG-HA-degron::LIN-29A::lin-29 3'UTR::operon linker (SL2) with GFP-H2B::tbb-2 3'UTR]* *lin-29(xe37)* II / mnC1 | This study |
| UR275 | *him-5(e1490) dmd-3(ok1327) V* | (Mason et al., 2008) |

Supplemental References:

Hodgkin J, Horvitz HR, Brenner S (1979) Nondisjunction mutants of the nematode Caenorhabditis elegans. Genetics 91: 67–94.

Lehrbach NJ, Armisen J, Lightfoot HL, Murfitt KJ, Bugaut A, Balasubramanian S, Miska EA (2009) LIN-28 and the poly(U) polymerase PUP-2 regulate let-7 microRNA processing in Caenorhabditis elegans. Nat Struct Mol Biol 16: 1016–1020. 10.1038/nsmb.1675
